# Angiographic Outcomes of Direct and Combined Bypass Surgery in Moyamoya Disease

**DOI:** 10.3389/fneur.2019.01267

**Published:** 2019-12-03

**Authors:** Peicong Ge, Xun Ye, Xingju Liu, Xiaofeng Deng, Jia Wang, Rong Wang, Yan Zhang, Dong Zhang, Qian Zhang, Jizong Zhao

**Affiliations:** ^1^Department of Neurosurgery, Beijing Tiantan Hospital, Capital Medical University, Beijing, China; ^2^China National Clinical Research Center for Neurological Diseases, Beijing, China; ^3^Center of Stroke, Beijing Institute for Brain Disorders, Beijing, China; ^4^Beijing Key Laboratory of Translational Medicine for Cerebrovascular Disease, Beijing, China; ^5^Beijing Translational Engineering Center for 3D Printer in Clinical Neuroscience, Beijing, China

**Keywords:** moyamoya disease, direct and combined bypass, risk factors, bypass patency, postoperative collateral formation

## Abstract

**Objective:** To identify associated risk factors for the angiographic outcomes after direct and combined bypass surgery in moyamoya disease (MMD).

**Methods:** All direct and combined bypass procedures performed from June 2009 to May 2015 were screened in this prospective cohort study. Patients who acquired presurgical and follow-up catheter angiography were included. Bypass patency and postoperative collateral formation were evaluated. Univariate and multivariate logistic regression analyses were performed to determine the influence factors for bypass patency and postoperative collateral formation.

**Results:** In total, 188 consecutive bypass procedures were included. After an 18-month median follow-up, the anastomosis patency rate was 88.3%. Postoperative collateral formation was associated with the patency of the anastomosis (Gamma = 0.891, *p* < 0.001). Multivariate logistic regression analysis showed that presence of hemorrhage (OR, 0.298; 95% CI, 0.125–0.709; *p* = 0.006) was associated with obstructed anastomosis. Among the 188 bypass surgeries, 125 (63.2%) hemispheres had good postoperative collateral formation and 85 (36.8%) had poor postoperative collateral formation. Multivariate logistic regression analysis showed that younger age at operation (OR, 2.396; 95% CI, 1.231–4.664; *p* = 0.010) was associated with good postoperative collateral formation, while the poor postoperative collateral formation was related to presence of hemorrhage (OR, 0.329; 95% CI, 0.143–0.758; *p* = 0.009) and dilated anterior choroidal artery (OR, 0.472; 95% CI, 0.240–0.929; *p* = 0.030).

**Conclusions:** This study has demonstrated that presence of hemorrhage predicts lower patency rates. Younger age at operation was associated with good postoperative collateral formation, while the poor postoperative collateral formation was related to presence of hemorrhage and dilated anterior choroidal artery.

## Introduction

Moyamoya disease (MMD) is a chronic cerebrovascular disorder, which is characterized by progressive occlusion of the bilateral distal internal carotid arteries, making for neurological/neurocognitive impairment and recurrent stroke (1, 2). Bypass surgery is considered to be the treatment for improving neurological/neurocognitive status and secondary stroke prevention in MMD patients (3, 4).

Over the years, hundreds of studies have examined the efficacy of bypass surgery in MMD patients (3–6). In these studies, angiographic outcomes are the decisive factor for the success of the intervention and quality of life of the patients (7, 8). Although clinical outcome of MMD has been well-documented (9, 10), a paucity study has monitored the angiographic outcomes following bypass using postoperative digital subtraction angiography (DSA) and identified the associated risk factors for the angiographic outcomes in MMD (11).

The identification of associated risk factors for the angiographic outcomes after direct bypass (DB) and combined bypass (CB) would help the surgeons to find out which MMD patient is more suitable for the bypass procedures. Therefore, we performed this prospective study to analyze the angiographic outcomes of bypass surgery for MMD.

## Materials and Methods

### Patient Data

Our previous trial was a single-center registry, prospective cohort study to evaluate the effects of different surgical modalities on the clinical outcome of MMD (12). Patients with moyamoya syndrome were ruled out. The report of the previous trial showed that CB and DB are more effective at preventing recurrent ischemic strokes than indirect bypass (IB), and there was no difference in preventing recurrent hemorrhage. Meanwhile, we designed a cohort study using the longitudinal data on patients allocated to the DB and CB. Patients who received pre-surgical and follow-up DSA were collected and reviewed. The study was approved by the institutional review board of Beijing Tiantan Hospital, Capital Medical University. Written informed consent for research purposes was obtained from all patients at admission.

### Surgical Modalities

To start with, all bypasses were performed by two surgeons (D.Z. and R.W.) with over 10 years of experience in cerebrovascular surgery. DB and CB were the favored surgical procedures for most patients at our stroke center, for they could be used in children, adult, ischemic, or hemorrhagic patients (13). DB was performed as end-to-side anastomosis of branch of the superficial temporal artery (STA) to cortical branches of middle cerebral artery (MCA). As for CB, two types of procedures have been chosen; the first one is combined DB and encephalodurosynangiosis (EDS) and the second one combined DB and encephaloduroarteriosynangiosis (EDAS). To be specific, for EDS, dura was cut in a radial fashion, inverted, and inserted underneath the bone edge of the craniotomy, while EDAS was a combination of EDS and STA branch sutured onto the brain surface. In the end, bypass patency was routinely examined using intraoperative indocyanine green angiography after anastomosis, and patients had routinely received computed tomography to detect postoperative hemorrhage or stroke on the first day after surgery.

### Radiological Evaluations

**Figure 1:**
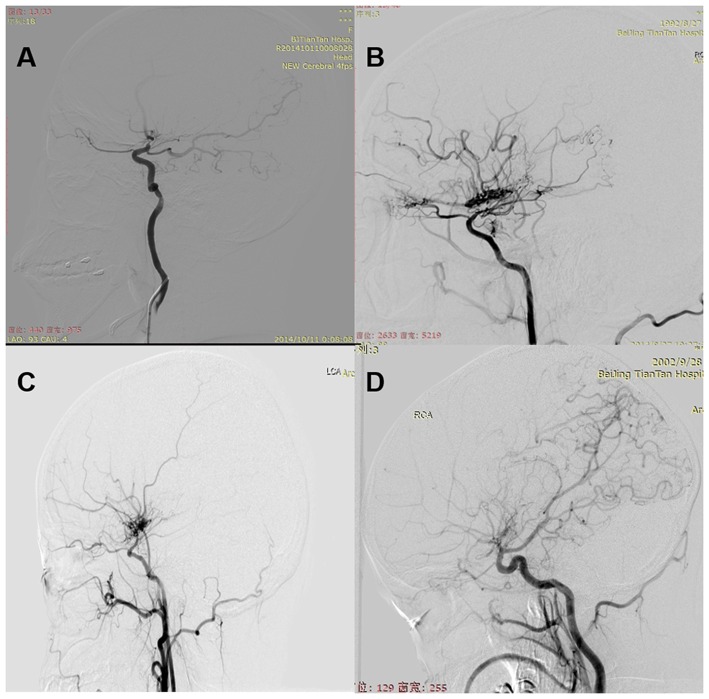
**(A)** Normal: normal AChA. **(B)** Dilated: dilated AChA with distal branching or abnormal branches. **(C)** Negative: normal or dilated PComA. **(D)** Positive: dilated PComA with abnormal branch extensions.

**Figure 2:**
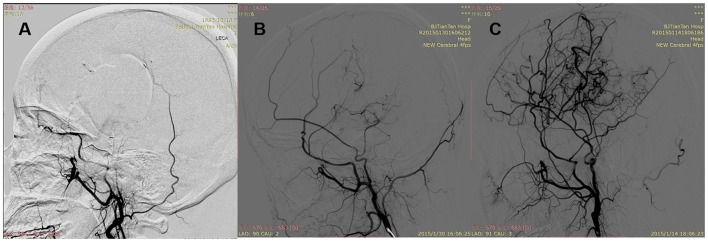
Patency of the anastomosis. **(A)** Occluded: complete proximal occlusion of STA and disappearance of MCA branches. **(B)** Stenosed: thin and stenosed STA with a few visible MCA branches. **(C)** Patent: patent or dilated STA with patent or even dilated MCA branches.

**Figure 3:**
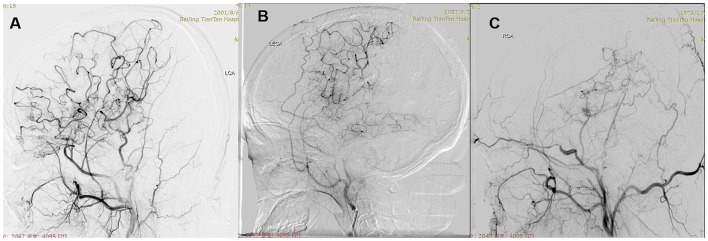
Postoperative collateral formation was evaluated with the Matsushima scale. **(A)** Level A: more than 2/3 of the MCA distribution. **(B)** Level B: between 2/3 and 1/3 of the MCA distribution. **(C)** Level C: slight or none.

The examination of computed tomography perfusion (CTP) was conducted for patients in no >6 months before surgical modalities (14). CTP parameters include regional cerebral blood volume (rCBV), regional cerebral blood flow (rCBF), mean transit time (MTT), and time to peak (TTP). The stages of preinfarction period were made as the following: Stage I, TTP was delayed, and MTT, rCBF, and rCBV were normal; Stage II, TTP and MTT were delayed, rCBF was normal, and rCBV was normal or slightly increased; Stage III, TTP and MTT were delayed, rCBF was decreased, and rCBV was normal or slightly decreased; Stage IV, TTP and MTT were delayed, and rCBF and rCBV were decreased. Preoperative and postoperative DSA were evaluated by two neurosurgeons and one radiologist, who were not involved in the surgery and blinded to clinical information. Preoperative DSA was performed at 1 month before surgery. Surgical hemispheres were evaluated based on Suzuki stage (15). The anterior choroidal artery (AChA) in surgical hemispheres was assessed (16): Normal, normal AChA; Dilated, dilated AChA with distal branching or abnormal branches. Posterior communicating artery (PComA) was recorded (16): Negative, normal or dilated PComA; Positive, dilated PComA with abnormal branch extensions (Figure 1). Follow-up DSA was performed at 6 to 12 months after surgery. Patency of the anastomosis at follow-up was evaluated (11): Occluded, complete proximal occlusion of STA and disappearance of MCA branches; Stenosed, thin and stenosed STA with a few visible MCA branches; Patent: patent or dilated STA with patent or even dilated MCA branches (Figure 2). Based on the above, Occluded and Stenosed were defined as “Obstructed” angiographic outcome and Patent was defined as “Unobstructed” angiographic outcome. Finally, postoperative collateral formation was evaluated with the Matsushima scale (17): Level A, more than 2/3 of the MCA distribution; Level B, between 2/3 and 1/3 of the MCA distribution; and Level C, slight or none (Figure 3). Based on the above, A and B were defined as “Good” angiographic outcome and C was defined as “Poor” angiographic outcome.

### Statistical Analysis

All statistical analyses were carried out using SPSS software (Windows version 22.0; IBM). The univariate and multivariate logistic regression model were used to identify which variables were associated with postoperative collateral formation and bypass patency. Odds ratios (OR) with 95% confidence intervals (CIs) are presented. A probability value < 0.05 was considered statistically significant.

## Results

### Baseline Characteristics

From June 2009 to May 2015, a total of 176 patients (188 hemispheres) at Stroke Center Ward 3 of our institution were included in this study. The median age at the operation was 34.5 years. The female/male ratio was 1.2:1.0. Among the 188 hemispheres, 47 (25.0%) had hypertension, 14 (7.4%) had a history of smoking, 9 (4.8%) had a history of alcohol use, 8 (4.3%) had diabetes, 6 (3.2%) had hyperlipidemia, and 2 (1.1%) had thyroid disease. The most common onset type was infarction (40.6%), followed by hemorrhage (29.0%), transient ischemic attack (TIA, 22.3%), frequent TIAs (≥2 times per month, 4.8%), headache (5.9%), seizures (2.1%), and no symptom (0.5%). Modified Rankin Scale score at admission lower than 2 was observed in 133 hemispheres (Table 1).

**Table 1:** Clinical characteristics of 188 hemispheres in this study.

**Characteristics**	**Total**
Hemispheres	188
Female/male ratio	1.2:1.0 (103/85)
Age at operation, median (IQR), y	34.5 (24.25–43.0)
Age	
<18 years	30 (16.0%)
**History of risk factors**	
Hypertension	47 (25.0%)
Smoking	14 (7.4%)
Diabetes	8 (4.3%)
Alcohol use	9 (4.8%)
Hyperlipidemia	6 (3.2%)
Thyroid disease	2 (1.1%)
**Clinical manifestation**	
Infarction	70 (37.2%)
Hemorrhage	51 (27.1%)
TIA	42 (22.3%)
Frequent TIAs	9 (4.8%)
Headache	11 (5.9%)
Seizures	4 (2.1%)
No symptom	1 (0.5%)
mRS >2 at admission	133 (70.7%)
**Suzuki stage**	
I	2 (1.1%)
II	29 (15.4%)
III	73 (38.8%)
IV	55 (29.3%)
V	26 (13.8%)
VI	3 (1.6%)
PCA involved	62 (33.0%)
AChA dilated	70 (37.2%)
PComA positive	19 (10.1%)
**Surgical modalities**	
DB	80 (42.6%)
DB+EDS	75 (39.9%)
DB+EDAS	33 (17.6%)
The stage of preinfarction period^*^	
Normal	6 (3.3%)
Stage I	7 (3.8%)
Stage II	39 (21.3%)
Stage III	84 (45.9%)
Stage IV	47 (25.7%)
DSA follow-up, median (IQR), mons	18 (12-35)

* *183 hemispheres received CTP*.

*AChA, anterior choroidal artery; DB, direct bypass; DB+EDS, direct bypass and encephalodurosynangiosis; DB+EDAS, direct bypass and encephaloduroarteriosynangiosis; DSA, digital substraction angiography; IQR, interquartile range; mRS, modified Rankin Scale; PCA, posterior cerebral artery; PComA, posterior communicating artery; TIA, transient ischemic attack*.

Most hemispheres presented with Suzuki Stage 3 (38.8%). Posterior cerebral artery (PCA) involved was observed in 62 (33.0%) hemispheres. Among 188 hemispheres, 70 (37.2%) had dilated AChA, and 19 (10.1%) had positive PComA. In addition, 80 (42.6%) hemispheres were treated with DB, 75 (39.9%) hemispheres received DB+EDS, and 33 (17.6%) hemispheres were treated with DB+EDAS.

### Angiographic Outcomes

During the 18-month DSA median follow-up, the distribution of patency of the anastomosis was as follows: Occluded, *n* = 22 (11.7%); Stenosed, *n* = 34 (18.1%); Patent, *n* = 132 (70.2%). The anastomosis patency rate was 88.3%. Among a total of 188 hemispheres, 69 (36.7%) achieved Matsushima level A, 56 (29.8%) achieved Matsushima level B, and 63 (33.5%) achieved Matsushima level C. Postoperative collateral formation was associated with the patency of the anastomosis (Gamma = 0.891, *p* < 0.001). It was notable that although the anastomosis was occluded, two hemispheres treated with DB+EDAS still achieved Matsushima level B (Table 2).

**Table 2:** Angiographic outcomes.

**Outcome**	**Total (*n* = 188)**	**Matsushima scale**			*p*-value^*^
		**Level A (*n* = 69)**	**Level B (*n* = 56)**	**Level C (*n* = 63)**	
Bypass patency					<0.001
Occluded	22 (11.7%)	0 (0.0%)	2 (3.6%)	20 (31.7%)	
Stenosed	34 (18.1%)	2 (2.9%)	7 (12.5%)	25 (39.7%)	
Patent	132 (70.2%)	67 (97.1%)	47 (83.9%)	18 (28.6%)	

* *Goodman-Kruskal Gamma test, Gamma = 0.891*.

### Analysis for Predictive Factors for Patency of the Anastomosis

Among the 188 hemispheres that have undergone bypass surgery, 132 (70.2%) hemispheres had unobstructed anastomosis and 56 (29.8%) had obstructed anastomosis. Univariate logistic regression analysis showed that the presence of hemorrhage (OR, 0.322; 95% CI, 0.143–0.721; *p* = 0.006) and the stage of preinfarction periods (OR, 1.392; 95% CI, 1.002–1.934; *p* = 0.049) affected patent bypass, and the other factors were found not significantly associated. Multivariate logistic regression analysis showed that presence of hemorrhage (OR, 0.298; 95% CI, 0.125–0.709; *p* = 0.006) was associated with obstructed anastomosis (Table 3). Furthermore, among 30 pediatric hemispheres, 24 (80.0%) had unobstructed anastomosis, and 6 (20.0%) had obstructed anastomosis. However, univariate logistic regression analysis did not find the factors affecting patent bypass (Supplemental Table 1). In addition, among 158 adult hemispheres, 108 (68.4%) had unobstructed anastomosis, and 50 (31.6%) had obstructed anastomosis. Univariate logistic regression analysis showed that the presence of hemorrhage (OR, 0.329; 95% CI, 0.135–0.801; *p* = 0.014) was basically the only factor that affected patent bypass, while the other factors were not significantly associated. The other factors were not significantly associated. Multivariate logistic regression analysis showed that presence of hemorrhage (OR, 0.313; 95% CI, 0.126–0.777; *p* = 0.012) was associated with obstructed anastomosis (Supplemental Table 2).

**Table 3:** Logistic regression analysis of predictors for bypass patency.

**Characteristics**	**Bypass patency**		***p*****-value**		**OR (95% CI)**
	**Unobstructed (*n* = 132)**	**Obstructed (*n* = 56)**	**Uni**	Multi^*^	
**Age, years**					
<35	24 (18.2%)	6 (10.7%)	0.339		
≥35	108 (81.8%)	50 (89.3%)			
Female sex	70 (53%)	33 (58.9%)	0.590		
**History of risk factors**					
Hypertension	34 (25.8%)	13 (23.2%)	0.069		
Smoking	10 (7.6%)	4 (7.1%)	0.918		
Diabetes	6 (4.5%)	2 (3.6%)	0.215		
Alcohol use	8 (6.1%)	1 (1.8%)	0.613		
Hyperlipidemia	4 (3.0%)	2 (3.6%)	0.485		
Thyroid disease	2 (1.5%)	0 (0.0%)	0.999		
**Clinical manifestation**					
Hemorrhage	28 (21.2%)	23 (41.1%)	**0.006**	**0.006**	0.298 (0.125–0.709)
Infarction	51 (38.6%)	19 (33.9%)	0.394	0.367	0.684 (0.299–1.563)
Others	53 (40.2%)	14 (25.0%)			
Suzuki stage			0.164		
I	0 (1.5%)	0 (0.0%)			
II	3 (12.9%)	12 (21.4%)			
III	10 (42.4%)	17 (30.4%)			
IV	10 (29.5%)	16 (28.6%)			
V	1 (12.1%)	10 (17.9%)			
VI	0 (1.5%)	1 (1.8%)			
AChA dilated	12 (34.1%)	25 (44.6%)	0.172		
PComA positive	3 (8.3%)	8 (14.3%)	0.227		
PCA involved	9 (31.1%)	21 (37.5%)	0.604		
The stage of preinfarction period			**0.049**	0.062	1.379 (0.984–1.932)
Normal	2 (1.6%)	4 (7.3%)			
Stage I	4 (3.1%)	3 (5.5%)			
Stage II	26 (20.3%)	13 (23.6%)			
Stage III	61 (47.7%)	23 (41.8%)			
Stage IV	35 (27.3%)	12 (21.8%)			
**Surgical modalities**					
DB+EDAS	22 (16.7%)	11 (19.6%)	0.368	0.096	0.447 (0.174–1.152)
DB+EDS	50 (37.9%)	25 (44.6%)	0.255	0.155	0.584 (0.279–1.346)
DB	60 (45.5%)	20 (35.7%)			

* *Adjusted for surgical modalities. One hundred and eighty-three hemispheres received CTP. Boldface indicates statistical significance (p <0.05)*.

*AChA, anterior choroidal artery; DB, direct bypass; DB+EDS, direct bypass and encephalodurosynangiosis; DB+EDAS, direct bypass and encephaloduroarteriosynangiosis; CI, confidence intervals; OR, odds ratios; PCA, posterior cerebral artery; PComA, posterior communicating artery*.

### Analysis for Predictive Factors for Postoperative Collateral Formation

Of the 188 bypass procedures, 125 (63.2%) hemispheres had good postoperative collateral formation and 85 (36.8%) had poor postoperative collateral formation. It could be found from the univariate logistic regression that age is related to the condition of postoperative collateral formation, for patients who had the operation (OR, 2.287; 95% CI, 1.226–4.264; *p* = 0.009) at a younger age had better postoperative collateral formation. On the other hand, the presence of hemorrhage (OR, 0.300; 95% CI, 0.136–0.664; *p* = 0.003) and dilated AChA (OR, 0.518; 95% CI, 0.278–0.963; *p* = 0.038) were identified as predictors of poor postoperative collateral formation (Table 2). Similarly, multivariate logistic regression analysis showed that age is related to the condition of postoperative collateral formation, for patients who had the operation (OR, 2.396; 95% CI, 1.231–4.664; *p* = 0.010) at a younger age had good (better) postoperative collateral formation, while presence of hemorrhage (OR, 0.329; 95% CI, 0.143–0.758; *p* = 0.009) and dilated AChA (OR, 0.472; 95% CI, 0.240–0.929; *p* = 0.030) were associated with poor postoperative collateral formation (Table 4). Besides, among 30 pediatric hemispheres, 25 (83.3%) had good postoperative collateral formation and 5 (16.7%) had poor postoperative collateral formation. Univariate logistic regression analysis did not find the factors that affected postoperative collateral formation (Supplemental Table 3). On the other hand, among 158 adult hemispheres, 100 (63.3%) had good postoperative collateral formation and 58 (36.7%) had poor postoperative collateral formation. Univariate logistic regression analysis showed the presence of hemorrhage (OR, 0.306; 95% CI, 0.128–0.731; *p* = 0.008) and dilated PComA (OR, 0.257; 95% CI, 0.080–0.832; *p* = 0.023) could be identified as predictors of poor postoperative collateral formation. Finally, the multivariate logistic regression analysis showed that only presence of hemorrhage (OR, 0.349; 95% CI, 0.141–0.861; *p* = 0.022) was basically the only factor that associated with poor postoperative collateral formation (Supplemental Table 4).

**Table 4:** Logistic regression analysis of predictors for postoperative collateral formation.

**Characteristics**	**PCF**		***p*****-value**		**OR (95% CI)**
	**Good (*n* = 125)**	**Poor (*n* = 63)**	**Uni**	Multi^*^	
**Age, years**					
<35	71 (56.8%)	23 (36.5%)	**0.009**	**0.010**	2.396 (1.231–4.664)
≥35	54 (43.2%)	40 (63.5%)			
Female sex	70 (56.0%)	33 (52.4%)	0.638		
**History of risk factors**					
Hypertension	29 (23.2%)	18 (28.6%)	0.183		
Smoking	9 (7.2%)	5 (7.9%)	0.444		
Diabetes	6 (4.8%)	2 (3.2%)	0.322		
Alcohol use	7 (5.6%)	2 (3.2%)	0.480		
Hyperlipidemia	3 (2.4%)	3 (3.2%)	0.391		
Thyroid disease	2 (1.6%)	0 (0.0%)	0.626		
**Clinical manifestation**					
Hemorrhage	26 (20.8%)	25 (39.7%)	**0.003**	**0.009**	0.329 (0.143–0.758)
Infarction	47 (37.6%)	23 (36.5%)	0.173	0.226	0.609 (0.273–1.359)
Others	52 (41.6%)	15 (23.8%)			
Suzuki stage			0.584		
I	2 (1.6%)	0 (0.0%)			
II	16 (12.8%)	13 (20.6%)			
III	51 (40.8%)	22 (34.9%)			
IV	41 (32.8%)	14 (22.2%)			
V	14 (11.2%)	12 (19.0%)			
VI	1 (0.8%)	2 (3.2%)			
PCA involved	43 (34.4%)	19 (30.2%)	0.941		
AChA dilated	40 (32.0%)	30 (47.6%)	**0.038**	**0.030**	0.472 (0.240–0.929)
PComA positive	9 (7.2%)	10 (15.9%)	0.072		
The stage of preinfarction period			0.438		
Normal	2 (1.7%)	4 (6.3%)			
Stage I	4 (3.3%)	3 (4.8%)			
Stage II	26 (21.7%)	13 (20.6%)			
Stage III	59 (49.2%)	25 (39.7%)			
Stage IV	29 (24.2%)	18 (28.6%)			
**Surgical modalities**					
DB+EDAS	23 (18.4%)	10 (15.9%)	0.921	0.387	0.655 (0.251–1.709)
DB+EDS	47 (37.6%)	28 (44.4%)	0.425	0.180	0.611 (0.298–1.254)
DB	55 (44.0%)	25 (39.7%)			

* *Adjusted for surgical modalities. One hundred and eighty-three hemispheres received CTP. Boldface indicates statistical significance (p <0.05)*.

*AChA, anterior choroidal artery; DB, direct bypass; DB+EDS, direct bypass and encephalodurosynangiosis; DB+EDAS, direct bypass and encephaloduroarteriosynangiosis; CI, confidence intervals; OR, odds ratios; PCA, posterior cerebral artery; PCF, postoperative collateral formation; PComA, posterior communicating artery*.

## Discussion

Since the 1970s, STA-MCA anastomosis has been used in MMD patients (18). Successful anastomosis between the donor and recipient arteries can improve blood flow immediately after surgery (4, 19). Bypass surgery contributes in improving neurological/neurocognitive status and secondary stroke prevention in MMD patients. Although the clinical outcomes of bypass surgery in MMD have been well-documented, there is lack of research on angiographic outcomes after bypass surgery for investigating the associated risks of MMD angiographic results using DSA (11). In this study, we performed this prospective study to investigate associated risk factors for the angiographic outcomes after bypass surgery. We found that younger age at operation was associated with good postoperative collateral formation, while the presence of hemorrhage and dilated AChA were associated with poor postoperative collateral formation. Meanwhile, the presence of hemorrhage was the only factor associated with obstructed anastomosis.

Bypass patency is an important determinant factor to evaluate the success of the surgery and the long-term outcome of the patient (8, 11, 20). Although the primary bypass function has been evaluated during surgery, there is a 4–10% chance of early bypass failure (21). Yoon et al. analyzed the long-term patency of 430 bypasses with postoperative imaging and found out that the overall patency rate of bypasses in MMD was 98% (8). The Caucasian Krupp Hospital cohort showed that, with the use of duplex ultrasound, the patency of the STA–MCA bypass at 3 months was 100% (22). In addition, Ha et al. showed that the postoperative patency of single barrel STA-MCA was 88.4% on follow-up imaging (mean, 16.5 months) (23). In our study, the patency of the bypasses was 88.3% on follow-up DSA (median, 18 months), which was similar with the study of Ha et al.

The presence of hemorrhage was the only factor associated with obstructed anastomosis. Yoon et al. conducted 430 consecutive bypasses, and their results revealed that low-flow bypass was associated with higher patency rate. Therefore, they speculated that MMD has high demand to augment blood flow, which encourages bypass patency (8). Interestingly, our recent study showed that hemorrhagic patients suffer less from hypoperfusion (14). Therefore, it is possible that ischemic MMD has higher demand to augment blood flow on the bypasses than the hemorrhagic MMD, which could lead to a conclusion that the hemorrhagic MMD was associated with obstructed anastomosis. However, only univariate logistic regression analysis showed that the stage of preinfarction periods was correlated with patent bypass and multivariate logistic regression analysis showed that preinfarction periods were unrelated to patent bypass. Furthermore, we found that postoperative collateral formation was associated with the patency of the anastomosis (Gamma = 0.891, *p* < 0.001). Our previous study also showed that bypass patency contributed to good angiographic outcome (11). It was noteworthy that although the anastomosis was occluded, two hemispheres treated with DB+EDAS still achieved Matsushima level B. Likewise, Kim et al. also demonstrated that clinical improvement of non-patent anastomosis can be expected after bypass surgery for adult MMD (20).

Despite the controversy, bypass is one of the main treatments of MMD for preventing recurrent stroke and to improve the prognosis (3, 4, 6, 10). The effect of bypass is based on postoperative collateral formation from the extracranial carotid artery into ischemic brain tissue (14, 24). Besides, multivariate logistic regression analysis showed that the patient who had the operation at a younger age (OR, 2.396; 95% CI, 1.231–4.664; *p* = 0.010) is more likely to have a better postoperative collateral formation. Previously reported studies had shown that bypass surgery is more effective in younger MMD patients (12). In addition, the presence of hemorrhage (OR, 0.329; 95% CI, 0.143–0.758; *p* = 0.009) was associated with poor postoperative collateral formation. Compared with the non-surgical group, the results of the Japan Adult Moyamoya Trial revealed that direct bypass undertook certain roles in preventing rebleeding (25). In general, despite aggressive bypass surgery, hemorrhagic MMD patients had higher morbidity and mortality than other types of MMD (26). For instance, in this study, only 28 (54.9%) of 51 had good postoperative collateral formation, which could be an explanation of the worse long-term clinical outcome in hemorrhagic MMD. Moreover, it is interesting for us to know that the dilated AChA (OR, 0.472; 95% CI, 0.240–0.929; *p* = 0.030) was also associated with poor postoperative collateral formation. It is well-known that the dilatation of AChA was a predictor of hemorrhage in MMD patients (16). A recent study showed that choroidal collaterals are associated with high rebleeding risk in non-surgical cohort and non-hemorrhagic hemispheres (27, 28). However, whether the dilated AChA is associated with poor postoperative collateral formation should be further verified.

DB is thought to provide immediate blood flow by the STA-MCA anastomosis. Meanwhile, indirect surgery takes more time to improve cerebral blood flow, which requires approximately about 3 months for neoangiogenesis from connective tissue (4, 19). DB provided early augmentation of blood flow, whereas the indirect surgery provided a more durable long-term neoangiogenesis, indicating a complementary association between the two procedures (29). Theoretically, CB may have better angiographic outcomes than DB. Many studies confirmed that DB and CB were more effective than indirect surgery in preventing recurrent stroke in adult (10, 30). However, for pediatric patients, indirect surgery can yield similar results with DB and CB (31), but there are few studies on superior surgical modality between DB and CB up until now. In our study, DB and CB had similar angiographic outcomes (postoperative collateral formation and bypass patency). Even so, we hold the opinion that abandoning CB for MMD is unwise, for the neoangiogenesis from indirect surgery might bring additional blood supply and remedy for patients who had STA-MCA anastomosis occlusion.

## Limitation

The present study had a few limitations. First, it is a non-randomized controlled study. Selection bias in choosing the bypasses (DB or CB) may exist. Second, this is a single neurosurgery center study, and referral and selection bias may exist. Third, not all patients have done follow-up DSA due to differences in medical conditions, which might lead to biased results. Besides, long-term follow-up DSA was not available. Therefore, we were unable to investigate the long-term angiographic outcomes of the bypasses. Further studies on long-term angiographic outcomes of the bypasses are needed to confirm our conclusions.

## Conclusion

This study has demonstrated that the presence of hemorrhage predicts lower patency rates. Besides, the study also found out the factors that affect the postoperative collateral formation. Younger age at operation was associated with good postoperative collateral formation, while presence of hemorrhage and dilated AChA were associated with poor postoperative collateral formation.

## Data Availability Statement

The datasets analyzed in this manuscript are not publicly available. Requests to access the datasets should be directed to PG, gepeicong@163.com.

## Ethics Statement

The studies involving human participants were reviewed and approved by IRB of Beijing Tiantan Hospital, Capital Medical University. Written informed consent to participate in this study was provided by the participants' legal guardian/next of kin.

## Author Contributions

PG and QZ: conception, design, analysis, and interpretation of data. PG, XY, XL, JW, and XD: acquisition of data. PG: drafting the article. RW, YZ, and DZ: technical support and surgery. JZ: approved the final version of the manuscript on behalf of all authors. JZ and QZ: study supervision. All authors critically revised the article and reviewed the submitted version of the manuscript.

### Conflict of Interest

The authors declare that the research was conducted in the absence of any commercial or financial relationships that could be construed as a potential conflict of interest.
