# Supplementary material for: Angiographic Outcomes of Direct and Combined Bypass Surgery in Moyamoya Disease
Source: Front Neurol. 2019 Dec 3;10:1267. doi: 10.3389/fneur.2019.01267 (PMC6903770; doi:10.3389/fneur.2019.01267)
Supplement: Supplementary file 1 [file Data_Sheet_1.pdf]

Supplemental Table 1. Subgroup analysis of predictors for bypass patency in pediatric patients.

Characteristics	Bypass patency		p value
	Unobstructed (n=24)	Obstructed (n=6)	Uni
Female sex	13(70.8%)	4(66.7%)	0.795
History of risk factors			
Hypertension	0(0.0%)	0(0.0%)	-
Smoking	0(0.0%)	0(0.0%)	-
Diabetes	0(0.0%)	0(0.0%)	-
Alcohol use	0(0.0%)	0(0.0%)	-
Hyperlipidemia	0(0.0%)	0(0.0%)	-
Thyroid disease	1(4.2%)	0(0.0%)	1.000
Clinical manifestation			
Hemorrhage	1(4.2%)	0(0.0%)	0.171
Infarction	6(25.0%)	3(50.0%)	0.901
Others	17(70.8%)	3(50.0%)	
Suzuki stage			0.854
I	0(0.0%)	0(0.0%)	
II	3(12.5%)	0(0.0%)	
III	10(41.7%)	5(83.3%)	
IV	10(41.7%)	1(16.7%)	
V	1(4.2%)	0(0.0%)	
VI	0(0.0%)	0(0.0%)	
AChA dilated	12(50.0%)	4(66.7%)	0.667
PComA positive	3(12.5%)	1(16.7%)	1.000
PCA involved	9(37.5%)	2 (33.3%)	0.808
Surgical modalities			
DB+EDAS	2(8.3%)	2(33.3%)	0.183
DB+EDS	11(45.8%)	3(50.0%)	0.212

DB	11(45.8%)	1(16.7%)

Abbreviations: AChA, anterior choroidal artery; DB, direct bypass; DB+EDS, direct bypass and encephalodurosynangiosis; DB+EDAS, direct bypass and encephaloduroarteriosynangiosis; CI, confidence intervals; OR, odds ratios; PCA, posterior cerebral artery; PComA, posterior communicating artery.

Supplemental Table 2. Subgroup analysis of predictors for bypass patency in adult patients.

Characteristics	Bypass patency		p value		OR (95% CI)
	Unobstructed (n=108)	Obstructed (n=50)	Uni	Multi*	
Female sex	57(52.8%)	29(58.0%)	0.474		
History of risk factors					
Hypertension	34(31.5%)	13(26.0%)	0.077		
Smoking	10(12.0%)	4(8.0%)	0.963		
Diabetes	6(5.6%)	2(4.0%)	0.196		
Alcohol use	8(7.4%)	1(2.0%)	0.641		
Hyperlipidemia	4(3.7%)	2(4.0%)	0.503		
Thyroid disease	1(9.3%)	0(0.0%)	1.000		
Clinical manifestation					
Hemorrhage	27(25.0%)	23(46.0%)	**0.014**	**0.012**	0.313 (0.126-0.777)
Infarction	45(41.7%)	16(32.0%)	0.373	0.342	0.652 (0.269-1.577)
Others	36(33.3%)	11(22.0%)			
Suzuki stage			0.124		
I	2(1.9%)	0(0.0%)			
II	14(13.0%)	12(24.0%)			
III	46(42.6%)	12(24.0%)			
IV	29(26.9%)	15(30.0%)			
V	15(13.9%)	10(20.0%)			
VI	2(1.9%)	1(2.0%)			
AChA dilated	33(30.6%)	21(42.0%)	0.228		
PComA positive	8(7.4%)	7(14.0%)	0.172		
PCA involved	9(31.1%)	19(38.0%)	0.666		
Surgical modalities					
DB+EDAS	20(18.5%)	9(18.0%)	0.841	0.156	0.512(0.203-1.290)

DB+EDS	39(36.1%)	22(44.0%)	0.759	0.251	0.657(0.321-1.346)
DB	49(45.4%)	19(38.0%)			

*Adjusted for surgical modalities. Boldface indicates statistical significance ($p < 0.05$).

Abbreviations: AChA, anterior choroidal artery; DB, direct bypass; DB+EDS, direct bypass and encephalodurosynangiosis; DB+EDAS, direct bypass and encephaloduroarteriosynangiosis; CI, confidence intervals; OR, odds ratios; PCA, posterior cerebral artery; PComA, posterior communicating artery.

Supplemental Table 3. Subgroup analysis of predictors for postoperative collateral formation in pediatric patients.

Characteristics	PCF		p value
	Good (n=25)	Poor (n=5)	Uni
Female sex	14(56.0%)	3(60.0%)	0.638
History of risk factors			
Hypertension	0(0.0%)	0(0.0%)	-
Smoking	0(0.0%)	0(0.0%)	-
Diabetes	0(0.0%)	0(0.0%)	-
Alcohol use	0(0.0%)	0(0.0%)	-
Hyperlipidemia	0(0.0%)	0(0.0%)	-
Thyroid disease	1(4.0%)	0(0.0%)	1.000
Clinical manifestation			
Hemorrhage	1(4.0%)	0(0.0%)	0.171
Infarction	7(28.0%)	2(40.0%)	0.155
Others	17(68.0%)	3(60.0%)	
Suzuki stage			0.486
I	0(0.0%)	0(0.0%)	
II	3(12.0%)	0(0.0%)	
III	11(44.0%)	4(80.0%)	
IV	10(40.0)	1(20.0%)	
V	1(4.0%)	0(0.0%)	
VI	0(0.0%)	0(0.0%)	
PCA involved	10(40.0%)	1 (20.0%)	0.349
AChA dilated	12(48.0%)	4(80.0%)	0.433
PComA positive	3(12.0%)	1 (20.0%)	0.583
Surgical modalities			
DB+EDAS	3(12.0%)	1(20.0%)	0.677

DB+EDS	11(44.0%)	3(60.0%)	0.831
DB	11(44.0%)	1(20.0%)	

Abbreviations: AChA, anterior choroidal artery; DB, direct bypass; DB+EDS, direct bypass and encephalodurosynangiosis; DB+EDAS, direct bypass and encephaloduroarteriosynangiosis; CI, confidence intervals; OR, odds ratios; PCA, posterior cerebral artery; PCF, postoperative collateral formation; PComA, posterior communicating artery.

.

Supplemental Table 4. Subgroup analysis of predictors for postoperative collateral formation in adult patients.

Characteristics	PCF		p value		OR (95% CI)
	Good (n=100)	Poor (n=58)	Uni	Multi*	
Female sex	56(56.0%)	30(51.7%)	0.447		
History of risk factors					
Hypertension	29(29.0%)	18(31.0%)	0.284		
Smoking	9(9.0%)	5(15.5%)	0.349		
Diabetes	6(6.0%)	2(3.4%)	0.263		
Alcohol use	7(7.0%)	2(3.4%)	0.401		
Hyperlipidemia	3(3.0%)	3(5.2%)	0.435		
Thyroid disease	1(1.0%)	0(0.0%)	1.000		
Clinical manifestation					
Hemorrhage	25(25.0%)	25(43.1%)	**0.008**	**0.022**	0.349(0.141-0.861)
Infarction	40(40.0%)	21(36.2%)	0.393	0.374	0.674(0.282-1.609)
Others	35(35.0%)	12(20.7%)			
Suzuki stage			0.385		
I	2(2.0%)	0(0.0%)			
II	13(13.0%)	13(22.4%)			
III	40(40.0%)	18(31.0%)			
IV	31(31.0%)	1322.4%)			
V	13(13.0%)	12(20.7%)			
VI	1(1.0%)	2(3.4%)			
PCA involved	33(33.0%)	18(31.0%)	0.677		
AChA dilated	28(28.0%)	26(44.8%)	0.078		
PComA positive	6(6.0%)	9(15.5%)	**0.023**	0.060	0.305(0.089-1.050)
Surgical modalities					
DB+EDAS	20(20.0%)	9(15.5%)	0.749	0.651	0.781(0.267-2.281)

DB+EDS	36(36.0%)	25(43.1%)	0.385	0.276	0.651(0.301-1.408)
DB	44(44.0%)	24(41.4%)			

*Adjusted for surgical modalities. Boldface indicates statistical significance ($p < 0.05$).

Abbreviations: AChA, anterior choroidal artery; DB, direct bypass; DB+EDS, direct bypass and encephalodurosynangiosis; DB+EDAS, direct bypass and encephaloduroarteriosynangiosis; CI, confidence intervals; OR, odds ratios; PCA, posterior cerebral artery; PCF, postoperative collateral formation; PComA, posterior communicating artery.

.
